# Dryland microbiomes reveal community adaptations to desertification and climate change

**DOI:** 10.1093/ismejo/wrae056

**Published:** 2024-03-29

**Authors:** Claudia Coleine, Manuel Delgado-Baquerizo, Jocelyne DiRuggiero, Emilio Guirado, Antoine L Harfouche, Cesar Perez-Fernandez, Brajesh K Singh, Laura Selbmann, Eleonora Egidi

**Affiliations:** Department of Ecological and Biological Sciences, University of Tuscia, Viterbo, 01100, Italy; Laboratorio de Biodiversidad y Funcionamiento Ecosistémico, Instituto de Recursos Naturales y Agrobiología de Sevilla (IRNAS), CSIC, Sevilla, E-41012, Spain; Department of Biology, Johns Hopkins University, Baltimore, MD 21218, United States; Department of Earth and Planetary Sciences, Johns Hopkins University, Baltimore, MD 21218, United States; Multidisciplinary Institute for Environment Studies “Ramón Margalef”, Universidad de Alicante, Alicante E-03071, Spain; Department for Innovation in Biological, Agro-Food and Forest systems, University of Tuscia, Viterbo 01100, Italy; Universidad Privada Boliviana, Cochabamba, Bolivia; Global Centre for Land-Based Innovation, Western Sydney University, Penrith 2750, Australia; Hawkesbury Institute for the Environment, Western Sydney University, Penrith 2750, Australia; Department of Ecological and Biological Sciences, University of Tuscia, Viterbo, 01100, Italy; Mycological Section, Italian Antarctic National Museum (MNA), Genoa 16128, Italy; Global Centre for Land-Based Innovation, Western Sydney University, Penrith 2750, Australia; Hawkesbury Institute for the Environment, Western Sydney University, Penrith 2750, Australia

**Keywords:** anthropogenic impact, climate change, dryland microbiomes, environmental drivers, drylands, extreme environments

## Abstract

Drylands account for 45% of the Earth’s land area, supporting ~40% of the global population. These regions support some of the most extreme environments on Earth, characterized by extreme temperatures, low and variable rainfall, and low soil fertility. In these biomes, microorganisms provide vital ecosystem services and have evolved distinctive adaptation strategies to endure and flourish in the extreme. However, dryland microbiomes and the ecosystem services they provide are under threat due to intensifying desertification and climate change. In this review, we provide a synthesis of our current understanding of microbial life in drylands, emphasizing the remarkable diversity and adaptations of these communities. We then discuss anthropogenic threats, including the influence of climate change on dryland microbiomes and outline current knowledge gaps. Finally, we propose research priorities to address those gaps and safeguard the sustainability of these fragile biomes.

## Introduction

**Figure 1:**
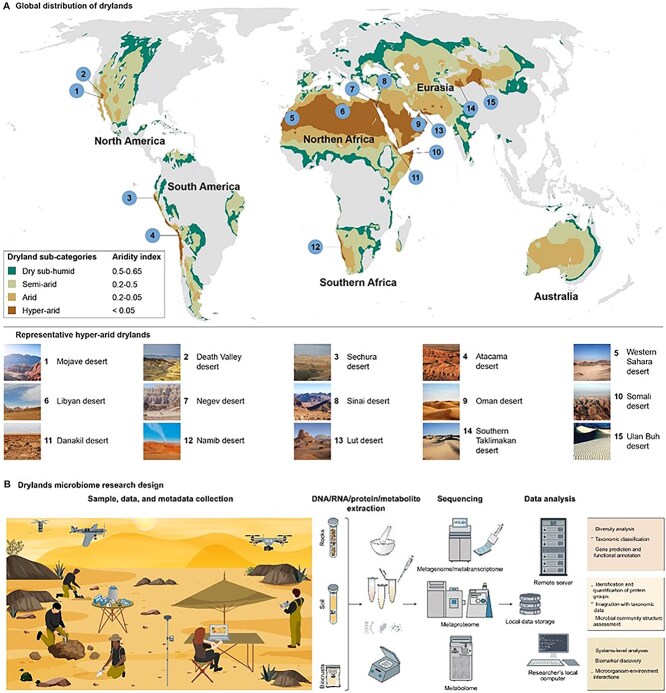
Global distribution and microbiome research design of drylands; (A) distribution of drylands worldwide according to the United Nations (UN) Convention to Combat Desertification and the Convention on Biological Diversity definitions using the aridity zone map created by the UN Environment Programme; drylands are categorized into four subgroups based on their aridity index; sites of 15 representative global hyperarid drylands are marked on the map (B); schematic illustration of drylands microbiome research, from designing the experiment to collecting time-series samples and high-resolution data, analyzing meta-omics datasets, and to providing insights into microbial ecosystems response to desertification and climate change; remote sensing platforms based on large and small satellite (CubeSat) constellations that work in unison as well as unmanned aerial vehicles (coordinated teams of drones) carrying imaging sensors (red–green–blue (RGB), multispectral, hyperspectral, thermal infrared, and light detection and ranging [LiDAR]) offer new ways to study dryland microbiome ecosystems and dynamics by providing spectroscopic and ranging data and capturing finer detail of biodiversity, including the microbial soil, biocrusts, and rocks inhabitants; microbiome data (metagenomics, metatranscriptomics, metaproteomics, and metabolomics) of soil, rocks, and biocrusts, generated by state-of-the-art high-throughput sequencing platforms, allow for rapid time course studies and large sample analyses; biological samples are labeled with quick response codes and information about each sample (when it was collected, where it was collected from, what kind of sample it is, and what were the environmental conditions at the time of sampling) and methods used in the experiments should be recorded in the experimental metadata; data and metadata are stored locally on a data storage device (e.g. a network attached storage), accessed by the researcher’s local computer, and transmitted to a remote server, with significant computing power, to analyze meta-omics data and their associated metadata.

Drylands are of paramount significance in their global distribution and the human population they support, regulating the global carbon (C), nitrogen (N), and water cycles [1]. Drylands constitute the largest terrestrial biome, covering about 45% of the global land surface and supporting almost half the world’s cultivated systems and half of its livestock [2], encompassing a plethora of ecosystems such as rangelands, grasslands, woodlands, savannahs, deserts, scrublands, and dry forests. The global geographic classification of drylands is often based on the aridity index and encompasses all regions where this index is between 0.05 and 0.65 (Fig. 1A). In addition to water scarcity, drylands, particularly arid and hyperarid systems, are characterized by polyextreme conditions [3, 4] such as high evaporation rates, extremely high solar ultraviolet (UV) radiation, and extremes of temperatures. Climate change and anthropogenic activities are making these challenging conditions even more extreme and unpredictable. Even small environmental changes could result in a severe alteration of the C balance, water availability, and the provision of multiple ecosystem services [5].

Drylands host a diverse array of microorganisms (hereafter dryland microbiomes) that can be free-living or symbiotic, associated with vascular plants, or within biological soil crusts (hereafter biocrusts). In drylands, microbiomes contribute to essential ecosystem functions, such as the formation of fertile islands, nutrient cycling, and climate regulation, while also providing the backbone for the ecological succession of vegetation in extreme regions [6]. Therefore, a potential loss of microbial diversity, and consequently loss of functionality, might dramatically compromise the productivity of dry regions.

Exploring dryland ecology, particularly belowground, and its response to climate change and other anthropogenic pressures is nowadays of primary importance. In this review, we provide a comprehensive synthesis of the most recent knowledge of the diversity and function of microbiomes inhabiting global drylands, although considering that there is a lack of data from several important deserts around the world. For example, major dry regions in North Africa, South America, and Eurasia are still undersampled and understudied [e.g. see literature [7, 8]]. Furthermore, we discuss the major climate-driven and anthropogenic threats to these key members of the ecosystem and adaptation strategies that might underpin microbial survival to the increasingly extreme conditions driven by climate change. We then outline a set of recommendations and directions that we hope will contribute to the design of more efficient conservation and restoration strategies to cope with increasing anthropogenic threats. The importance of vegetational attributes to dryland is not covered since it has been reviewed elsewhere (e.g. Maestre *et al.* [9]) and our review primarily focuses on studies from natural systems within drylands. However, we recognize that the overall dryland ecosystem, extending beyond wild areas, encompasses land use types (e.g. farming) and niches (e.g. fertility islands), which contribute to the definition of microbiomes in dryland ecosystems; however, these niches, despite their significance, fall outside the scope of our current analysis.

## Dryland microbiomes

Top soils harbor the majority of dryland microbes, where they can exist as free-living assemblages and in association with adapted vascular plants. However, in the most arid eco-regions, they form interconnected assemblages known as biocrusts, which are typically dominated by phototrophs cyanobacteria and chlorophycean algae [10]. The development of biocrusts and soil heterotrophic communities results in enhanced soil stability (e.g. via the release of exopolysaccharides), nutrient concentration, and water retention, which ultimately favors the growth and development of vascular plants [9].

Drylands also support microbial communities specialized in the colonization of lithic substrates. Particularly in hyperarid regions, rocks provide a physical structure and porous substrate, high water retention potential, and access to micronutrients (e.g. rock minerals), forming epilithic, hypolithic, or endolithic assemblages [6]. Epiliths and hypolithic are observed in drylands of almost all aridity classes; lichens, in particular, provide an attractive niche for a multitude of microorganisms, constituting hotspots for microbial diversification [11]. In hyperarid regions, endoliths dominate and represent the utmost dryland specialists [12].

### Bacteria and archaea

**Figure 2:**
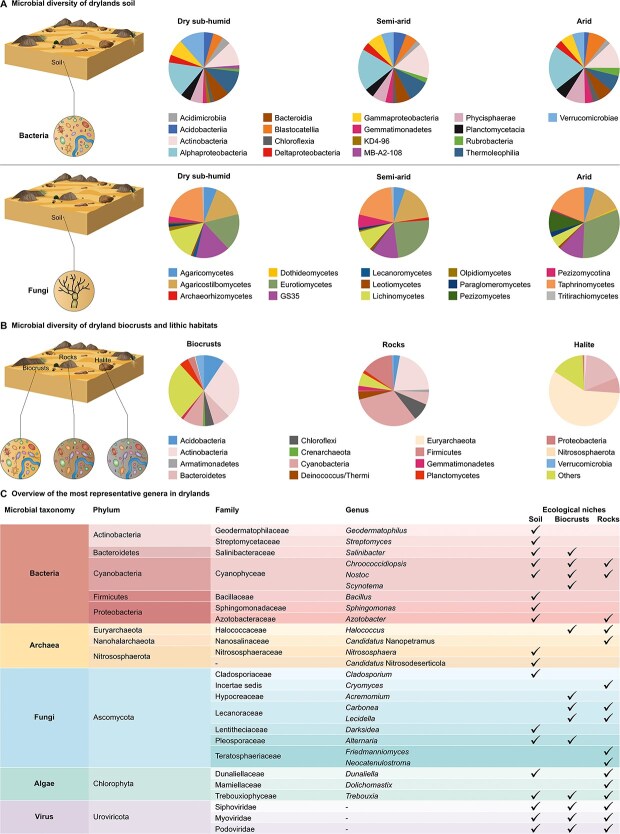
Overview of drylands microbiome diversity; (A) relative abundances of different bacterial and fungal groups at the global scale; relative abundances were calculated based on the number of reads retrieved by amplicon sequencing (16S ribosomal RNA [rRNA] gene and internal transcribed spacer [ITS] regions for bacteria and fungi, respectively); 16S rRNA and ITS datasets were obtained from soil global datasets [8, 83], which include samples collected in drylands ranging from subhumid to arid regions (Spain [Europe]), China, Australia, North Africa, North America, and South America); (B) most abundant microbial taxa found in biocrusts, rocks, and halites based on data from [14, 21, 59, 67, 76, 84–87]; data are presented from samples collected in dry regions worldwide, including Colorado Plateau (Colorado) Mojave Desert and Joshua Tree National Park (California), Canadian Arctic (Canada), Victoria Land (Continental Antarctica), Atacama Desert (Chile), Negev Desert (Israel), Northern Cape Province (South Africa), Libyan Desert, Australia, and Taklimakan Desert and Tibetan Plateau (China) ; (C) microbial taxonomy, phylum, family- and genus-level assignments, and ecological niches of the most notable examples of dryland microorganisms; hyphens (-) mean that the taxonomy classification of sequences is not available at a given level; KD4-96, uncultured *Chloroflexi*; MB-A2-108, uncultured *Actinobacteria*; GS35, uncultured *Ascomycota*.

Globally, members of *Actinobacteria*, *Alphaproteobacteria*, and *Chloroflexi* [13] dominate drylands (Fig. 2A and C). In soil, the dominant bacterial taxa belong to *Streptomyces mirabilis*, *Geodermatophilus obscurus*, as well as species from genera *Microcoleus*, *Phormidium*, *Plectonema*, *Schizothrix*, *Nostoc*, *Modestobacter*, and *Sphingomonas* [8]. In contrast, N-fixing bacteria are more abundant in biocrusts from hot drylands and include *Proteobacteria*, *Azospirillum* spp*.*, Deinococcus-Thermus *Deinococcus* spp., and cyanobacterial *Calothrix* spp*.* [14] (Fig. 2B). Conversely, endolithic bacterial communities harbor more generalist members of *Actinobacteria* (e.g. *Rubrobacter*, *Propionibacterium*, and *Solirubrobacter*),* Proteobacteria* (e.g. *Craurococcus*), and *Cyanobacteria* (e.g. *Chroococcidiopsis*) phyla.

Shotgun metagenomics has advanced our knowledge of the functional potential and evolutionary pathways of microorganisms in these extreme environments, revealing how drylands, and in particular hyperarid soils, are a reservoir of new microbial entities harboring potentially novel functional genes. Recent studies from the Atacama and Antarctic Deserts revealed that the majority of dryland-associated microbial genomes lack representatives in genomic public databases [13]. For example, the majority of new species of Antarctic endoliths were found to belong to monophyletic bacterial clades that diverged from related taxa 1.2 billion to 410 million years ago [15].

Among archaea, members of *Euryarchaeota* comprise the majority of sequences in highly saline soils/rocks. In particular, haloarchaea are typically associated with high salt in soils and halites [16]. A recent global survey of dryland soils showed that small-scale heterogeneity induced by plants, rather than large-scale changes in environmental conditions (e.g. soil pH), regulates the diversity, abundance, and co-occurrence network of nitrifying archaeal communities, which also include taxa with adaptation to energy starvation and extreme conditions [17].

### Fungi and algae

High-throughput sequencing and cultivation approaches showed a high level of fungal diversity in dry systems, inscribing these organisms among the most stress-tolerant eukaryotic life forms on Earth [18–20]. Globally, the soil dryland mycobiome is dominated by *Ascomycota*, followed by *Basidiomycota*, *Glomeromycota*, and *Zygomycota*, with *Alternaria*, *Fusarium*, *Chaetomium*, and *Cladosporium* as predominant genera. UV light, seasonality, and sand content have been identified as the most environmental determining critical shifts in community composition [20]. In contrast, our knowledge of biocrusts and rock fungal diversity and function is still limited. Indeed, previous studies have mainly focused on scattered localities (e.g. Qu *et al*. [21]), while a systematic view of these mycobiomes at a global scale is still lacking.

Especially in plant-free arid and hyperarid regions, oxygenic photosynthetic organisms are key primary producers. Such keystone organisms channel energy into the synthesis of energy and carbon storage compounds or are known to accumulate energy reserves in response to water stress or during the transition to dormancy. Among phototrophs, *Cyanobacteria* are typically the dominant group (e.g. *Chroococcidiopsis* and *Nostoc*). However, also green algae are widespread in drylands. The *Trebouxia genus* (family *Trebouxiophyceae*) and, to a lower extent, the *Chlorophyceae* family dominate [21]. In hypersaline systems, such as halite endoliths of the Atacama Desert, a unique alga of the *Dolichomastix* genus was shown to significantly contribute to the photosynthesis and the organic C budget of the community [22].

### Viruses

Viruses are considered the most abundant biological entities on Earth, with high genomic diversity and ecological and biogeochemical significance [23, 24]. Recent efforts led to important advances in reporting uncharacterized lineages (e.g. cyanophages) in hyperarid deserts [25]. Recent studies reported on the extent of the rock-associated virome. For example, a recent study reported transcriptionally active viruses of the order *Caudovirales* and the families *Pleolipoviridae* and *Sphaerolipoviridae* in halite endoliths of the Atacama Desert [22]. Analysis of putative extreme-tolerance genes and auxiliary metabolic genes provided evidence for a complex trade-off between viral predation and viral delivery of extreme-tolerance genes to their hosts, thereby aiding in their survival [24, 26]. Yet, study from Antarctic rocks [27] indicated high diversity but largely undescribed and spatially structured communities.

Overall, new technologies have generated unprecedented amounts of sequencing data giving us an ecosystem-wide view of the diversity, function, and biogeography of the global dryland microbiomes (Fig. 1B). However, this wealth of data has uncovered several knowledge gaps that require further investigation. For example, we have just begun to comprehend the amplitude of microbial diversity in drylands with most of them remaining undescribed. As such, extensive sampling studies are needed to describe the diversity, distribution, and drivers of different microbial taxa. In particular, we have little understanding of the interactions between the different life domains (e.g. bacteria and fungi; bacteria and algae). In addition, studies should encompass more remote and less explored dryland areas and neglected ecological niches (e.g. rocks) to capture the full spectrum of microbial diversity. Moreover, the existing data predominantly focus on bacterial and fungal communities, leaving a gap in our knowledge of other microorganisms and their roles in these ecosystems. For example, viral contributions at the ecosystem level, including the role of viruses in nutrient cycling and energy flow, spatial and temporal changes in biogeography, and interactions with host populations remain poorly understood. Similarly, large-scale surveys of microbes from higher taxa (e.g. protists) are still missing from drylands, although micro-eukaryotes are abundant in arid ecosystems and show sensitivity to aridity on a global scale [28].

## Impact of climate and anthropogenic drivers of change on dryland microbiomes

**Figure 3:**
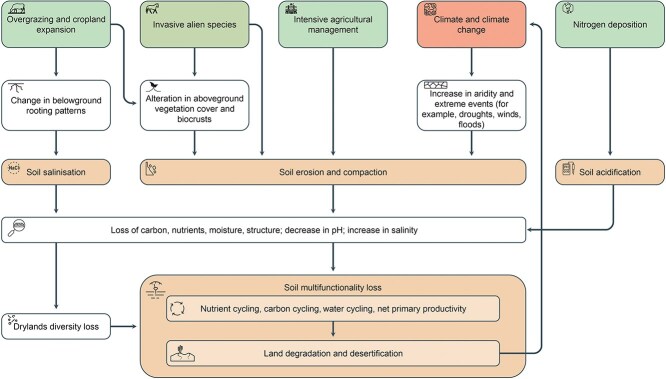
Drivers of land degradation and desertification in drylands; dryland soils are vulnerable to a range of physical (e.g. increase in aridity, droughts, winds, and floods), human-induced (e.g. nitrogen deposition via fertilization, overgrazing and intensive cropping), and biotic (e.g. invasive alien species) disturbances; these disturbances increase the rate of soil salinization, erosion, and compaction, whose consequences include both direct effects (e.g. shifts in microbial community composition and biodiversity loss) and indirect impacts via changes in physico-chemical soil properties (e.g. loss of carbon, nutrients, moisture, structure, decrease in pH, and increase in salinity); if not properly managed, these pressures can result in land degradation and, in arid areas, desertification, which will further accelerate the rate of climate change; through cascading effects, the drylands microbiome can ultimately impact ecosystem health and functioning.

The main drivers of dryland microbial dynamics could be broadly categorized into factors related to global climate (e.g. precipitation and temperature) and human activities (e.g. grazing, afforestation/deforestation, agriculture, fire management, urbanization, greenhouse gas emission) [5] (Fig. 3). The consequences of these pressures on the drylands ecosystem functioning include both direct effects (e.g. shifts in microbial community composition, biodiversity loss) and indirect impacts via changes in physico-chemical soil properties (e.g. losses in C, nutrients, moisture, structure, decrease in pH, increase in salinity) [29]. Given the fundamental role of microorganisms in supporting dryland functioning, microbial changes associated with climate change and physical disturbance can result in important fertility and multifunctionality loss that may lead to land degradation and desertification. It is estimated that 25%–35% of drylands are already degraded, with over 250 million people directly affected and about 1 billion people in over 100 countries at risk [30].

### Community responses to aridification

Aridity is a major climatic driver of ecosystem structure and functioning in drylands [31]. Recent studies indicate that aridity has increased globally during recent decades and is projected to increase significantly in the future due to changes in the amount and variability of precipitation, combined with increasing temperature and elevated atmospheric CO_2_, ultimately supporting the expansion of drylands [32]. These aridification processes can dramatically affect the soil microbiome of dry ecosystems, due to reduction in vegetation cover and decrease in N and C concentration [31] (Fig. 3). In fact, recent work suggests that aridity thresholds regulate ecosystem function in drylands, and that increases in aridity can negatively impact most microbial taxa and shift the compositional balance of key community members [33]. In particular, even small intensifications in aridity levels can result in drastic increases in the proportion of animal pathogens and reductions in the proportion of ectomycorrhizal fungi, soil C sequestration, and plant cover [31, 34]. Recent evidence also indicates that aridity thresholds can influence multiple physiological (e.g. soil microbial metabolic activity and biomass carbon) and functional (nitrogen cycling and multifunctionality) properties at the regional scale [34], while having drastic impacts on the complex functional interactions between microbial species within soil ecological networks and their multifunctionality [29, 35]. This overall effect can be further exacerbated at the local scale. For example, field experiments have found evidence that drought impacts the community structure and activity of soil microorganisms in drylands from two continents, with stronger responses detected in more mesic sites [36].

Global drying trends are also likely to impact complex biotic communities such as those of biocrusts found in arid and hyperarid systems. Experimental manipulation of climate has evidenced rapid mortality of dominant biocrust species, resulting in a total collapse of the surface biocrusts communities, and models concur that a warmer, drier future will generally reduce the abundance and the rate of C fixation in biocrusts [37]. Such warming-induced changes could reduce the physical stability of surface soils and their C storage potential. However, despite their vulnerability, biocrusts are critical for regulating the responses of multifunctionality to climate change and nutrient availability in arid ecosystems [38]. A recent global soil survey provided evidence that soil microbial communities largely regulate the impacts of increases in temperature, wetting-drying cycles, and nutrient additions on ecosystem multifunctionality [39].

### Functional responses to aridification

Aridity can also impact the functional potential and adaptation strategies of dryland communities. Dry conditions are challenging for microorganisms, which may suffer from loss of water, damage to cellular membranes, accumulation of reactive oxygen species, protein denaturation, and DNA damage. Survival in increasingly stressful environments requires specialized mechanisms of adaptation (Box 1), and along aridity gradients, soil communities are progressively enriched with genes related to these stress tolerance (e.g. DNA damage repair, cation transportation, sporulation, and osmolyte biosynthesis), harbor smaller and simplified genomes [40], and reduced community stability and interactions [23]. Consequently, increases in the incidence of stress tolerance/resource scavenging traits and simplified co-occurrence patterns are likely outcomes of community-level adaptations to increasing water-depletion driven by global change. For example, experimental drought in Mediterranean-like grasslands of California has been found to trigger the upregulation of transporter-related genes, as well as biosynthesis of cell membrane and wall compounds, and compatible solutes [41]. However, under climate change, cold drylands, especially those from arid (e.g. Eurasian steppe) or hyperarid regions (e.g. Antarctic, Arctic), are most likely to experience not only an intensified drought-induced aridity but also a large degree of warming during the cold season. Such processes have been linked to microbial changes such as increased microbial biomass, community complexity, and metabolic potential for nitrogen assimilation [42]. Consequently, shifts in the balance and occurrence of functional attributes could be fundamentally different across hot and cold drylands, but investigations comparing these contrasting biomes are currently lacking.

BOX 1: ENDURING THE EXTREMES: MAJOR ADAPTATIONS OF DRYLAND MICROORGANISMS TO HYPERARID CONDITIONS.

Arid and hyperarid regions are characterized by poly-extreme conditions (e.g. low water availability combined with temperature fluctuations, salinity, and high UV radiation) that require specialized mechanisms of adaptation. Microbial dryland specialists can produce extracellular polymeric substances (EPSs) to retain water and nutrients, and synthesize membranes rich in unsaturated fatty acids to maintain structural integrity [64]. They also support important membrane proton and cation pumps that allow them to survive under high pH environments [8]. Similarly, one of the major responses to harmful solar radiation is the production of UV-absorbing/screening pigments such as carotenoids, scytonemin, mycosporine-like amino acids, and melanin in fungi [65]. Microorganisms can also accumulate compatible solutes in their cytoplasm to combat osmotic stress [7] (Fig. Box 1). However, de novo production of compatible solutes is energetically expensive; thus, microorganisms have evolved transport systems to uptake available solutes produced by other community members, such as osmoprotectant uptake (Opu) proteins [66]. Yet, at the highest salt concentration, microorganisms, typically haloarchaea and a few bacteria, accumulate potassium chloride to balance the high osmotic pressure of their environment [67]. To minimize cellular damage, some organisms such as the endolithic *Chroococcidiopsis* have evolved both the capacity to convert photosynthetically active solar radiation into chemical energy using a complex molecular machinery and a number of acclimation and adaptive mechanisms including orange carotenoid protein and changes in the overall composition of their photosynthetic apparatus [68]. To counteract a water-stressed environment, fungi accumulate trehalose and intracellular glycerol as compatible solutes, which protect cell membranes from desiccation and freezing damage [69]. Constitutively melanized fungi have also evolved numerous morphological and osmotic adaptations to survive extremely saline conditions such as meristematic growth, pigmentation (melanin in cell walls), and changes in membrane composition and fluidity.

**Nutrient/energy sources**

Because of the scarcity of nutrients in hyperarid drylands, microbial communities are almost exclusively supported by the primary production of cyanobacteria and algae (i.e. autotrophs vs. heterotrophic communities). A mean to obtain energy for heterotrophs is the light-driven proton pump, bacteriorhodopsin, used by haloarchaea to augment their adenosine triphosphate (ATP) budget; a similar light-harvesting system, xanthorhodopsin, is found in the bacterium *Salinibacter* [70]. In drylands, atmospheric chemosynthesis supplements photosynthetic primary production in cold desert soils in the high Arctic, Antarctica, Tibetan Plateau, and South Australian desert, with trace gas oxidation providing the energy and/or carbon needed to sustain terrestrial ecosystems. Atmospheric hydrogen (H_2_) oxidation via [NiFe]-hydrogenases, as a means to harvest electrons, and the formation of ribulose-1,5-biphosphate carboxylase/oxygenase, have been reported for a broad range of bacteria, including members of the *Actinobacteria*, *Acidobacteria*, as well as *Proteobacteria* Ca. *Eremiobacterota* and Ca. *Dormibacterota* [71, 72]. These processes are thought to play an essential role in overcoming C and nutrient starvation in hyperarid deserts with this *in situ* metabolic hydrogenesis in specialized niche habitats may make a significant contribution to water availability and, therefore, to water activity and metabolic capacity [73].

The acquisition of N is also essential for dryland communities. In biocrusts, heterocyst cyanobacteria, particularly *Nostocales* and *Oscillatoriales*, dominate, actively fixing N and emitting nitric oxide and nitrous oxide [74]. Field experiments revealed that Antarctic communities had significant nitrogenase activity, suggesting that they play an important role in nutrient cycling in Antarctica soils [75]. It is thought that the high atmospheric deposition of nitrate over millions of years, as a result of photochemical processes in the upper atmosphere, most likely provides enough N to microbial communities of the desert. Indeed, pathways for uptake and ammonia assimilation have been identified in metagenome-assembled genomes from gypsum communities, indicating that deposited ammonia and nitrates might be major sources for N [76]. At the functional level, the most abundant community members of Antarctic soil are metabolically versatile aerobes, using ubiquitous atmospheric trace gasses to persist in a dormant state, meeting their energy and hydration needs via metabolic water production [13, 16]. The same strategy was found also crucial in endolithic microbiomes from Antarctica, whose active metabolism is, as average, limited to 1000 h per year [77].

**Ecological adaptations**

In addition to stress tolerance and resource-mining traits, it has been proposed that cooperative interactions (e.g. metabolic interdependence, cross-feeding relationships [78]) might be enhanced to support microbial survival in response to restrictive conditions such as those encountered in arid environments (e.g. oligotrophy) [78, 79]. These interactions support the survival of community members via mechanisms such as sharing of microbial byproducts and contribute to the functioning and stability of their ecosystems [79]. Such complex interactions are likely to establish during “windows of opportunity” (i.e. a short time window in which environmental conditions drop long enough below the hostile average level) allowing the organism enough time to develop tolerance and transition into more stable existence [80]. Yet, while being considered a potential driving force for microbial differentiation in important hyperarid communities, such as those inhabiting rocks [81], cooperative relationships have been directly observed in a relatively limited number of communities (e.g. biocrusts) [82] and further manipulative studies and field surveys are required to corroborate their role as an adaptation strategy in hyperarid microbiomes.

### An integrated multi-omics approach

Overall, although the existence of adaptation traits, and traits trade-offs, has been postulated for many microbial communities, studies focusing on trait-level consequences of climate change are still scarce in drylands. This is mostly because attempts to match taxonomic, physiological, and functional profiles are mostly hampered by the limitation of gene target amplicon sequencing, and the lack of a complete characterization of both taxonomic groups and functional genes, especially from arid communities. Filling these gaps will require efforts from multiple disciplines, including microbiology, ecology, bioinformatics, and artificial intelligence (AI) to isolate, identify, and characterize microbial species that inhabit global drylands. A systematic investigation encompassing multi-omics approaches (metagenomics, metabolomics, metaproteomics), culturing and *in situ* (e.g. using NanoSims) functional activity assessments, will be essential to (i) provide a more holistic understanding of eco-evolutionary mechanisms of adaptation and metabolic potential of microbiomes in dry environments and (ii) unravel various co-existing energy acquisition pathways point to diverse niches and the exploitation of available resources. Explicit consideration of environmental conditions (e.g. arid vs hyper arid) and niche specialization (e.g. soil vs rock vs vegetation) will provide valuable insights into the abilities of microorganisms to survive and function in dry conditions and their significance in maintaining the ecological balance of these regions worldwide. Additionally, most studies to date have focused on short-term responses to drought and warming, but it is unclear how microbial communities will respond to prolonged or permanent aridification processes. Long-term studies are needed to understand how microbial communities will adapt to changing environmental conditions, and how these adaptations will affect soil health and ecosystem functioning over time. In fact, shifts in community functional balance mediated by climate change might potentially result in functional changes, with feedback on biogeochemical cycles [43]. These associations might be particularly important for the most arid regions of the world, where effects of soil microbial diversity on ecosystem multifunctionality (e.g. via increasing organic matter decomposition and nutrient transformation) are expected to prevail due to lack of vegetation cover [35]. Overall, approaches that are strongly grounded in community-aggregated traits frameworks (e.g. the Y–A–S life history triangle) [41] show strong potential to help linking future climate systems to the intrinsic ability of these communities to withstand and recover from disturbance, and perform ecosystem functions.

### Brown or green? Dryland greening as an understudied global driver

Although the expansion of arid subtypes that become drier comprises the major outcome of global change, there have been global trends in dryland greening (i.e. significant increases in live green vegetation cover) at both the global and local scales [44]. The main drivers of this phenomenon have been attributed to global increased atmospheric CO_2_ levels (CO_2_ fertilization), which improve vegetation water-use efficiency and, consequently, increase soil moisture [45], as well as agricultural practices (e.g. irrigation) at the local scale [46]. Such shifts in vegetation and water dynamics could significantly impact microbial communities in these systems. For example, greater vegetation cover and establishment of fertility islands can result in an increase in plant litter and root exudates, which serve as a source of organic matter for microbes. This can lead to higher microbial biomass and diversity, which can support more complex food webs and ecosystem processes, while also favoring microbial communities that depend on labile carbon as a source of energy (i.e. copiotrophs) at the expense of those adapted to low levels of nutrients (i.e. oligotrophs). Critically, increases in microbial activity and respiration rates due to higher atmospheric CO_2_ concentrations can outpace the CO_2_ fixation rate mediated by plants, further reinforcing CO_2_ losses [47]. Yet, increases in aridity may limit the positive influence of CO_2_ and warming on plant productivity [48]. Further experimental evidence is needed to understand the specific impacts of vegetation cover and moisture increases on microbial community composition and function, and the implications of these potential changes for microbial-mediated ecosystem processes and services in drylands.

### Climate change and local drivers of change

Dryland microorganisms are highly vulnerable to physical disturbance both of natural and anthropogenic nature. For example, they are known to be highly influenced by insects (e.g. ant nests) and mammal disturbance. Similarly, microorganisms are vulnerable to cropping and grazing by livestock [49]. Crop cultivation and grazing accelerate erosion rates above natural levels by reducing natural soil-stabilizing covers, such as native vegetation and biocrusts, resulting in reduced bacterial biomass and shifts in the abundance of dominant bacterial populations [50]. Changes in the dominance of microbial taxa associated with grazing intensity can further influence the overall soil biodiversity and function of drylands. This effect has been worsened by the adoption of management practices such as tillage, which disrupts soil structure, accelerating surface runoff and topsoil C loss [51]. In some regions of the world, such as Australia, introduction and encroachment of invasive species (e.g. feral horses) have further contributed to soil erosion both directly, by removing surface soil, and indirectly, by causing declines in plant cover, biomass, and abundance [52]. Reduction in soil surface stability results in higher levels of soil movement, and thus increases in soil erodibility and soil displacement by wind and water (Fig. 3).

Poor management practices are also responsible for increased soil acidification rates in drylands, with deposition and leaching of N-based inorganic fertilizers being among the major contributors to this process. During acidification, soils undergo various pH buffer ranges associated with the weathering and liberation of different elements/constituents, such as soluble aluminum, which can affect root growth by restricting access to water and nutrients. Decreases in pH can also decrease soil microbial activity and diversity, affecting agriculturally important associations, such as mycorrhizae and N fixing bacteria [53].

The combination of climatic, anthropogenic, and biotic processes ultimately results in soil destabilization, nutrient depletion, and modified soil communities, consequently changing the functional capabilities of soil in relation to C and N cycling, decomposition, and plant growth [54]. As dryland microorganisms also contribute to global climate regulation through CO_2_, reactive N, and methane (CH_4_) emissions, these processes will also likely alter the rate of greenhouse gasses release relative to uptake, driving positive feedback and further accelerating the rate of climate change. Empirical evidence is emerging in support of the complex interactions between global and local factors and their potential impact on soil biota and the processes they mediate. For example, a recent global study has evidenced that increasing grazing pressure reduced ecosystem service delivery in warmer and species-poor drylands, whereas positive effects of grazing were observed in colder and species-rich areas [55]; similarly, herbivore exclusion in grasslands led to greater microbial metabolic quotient (respiration rate/biomass unit) only at sites with lower soil organic C (< 1.7%) [56].

Despite these clear linkages, significant knowledge gaps exist in our understanding of the interplay between local drivers of change and climatic factors on soil microbial communities and the biogeochemical processes they mediate. This is mainly because different drivers of change are often studied independently, especially when considering microbial communities, although most terrestrial communities are exposed to different physical, chemical, or biotic stressors that can move them out of their normal operating range. In addition, there is a limitation in current studies that explore the relationship between microbial composition and the environment at different scales. For example, global data overlook the intricate variations in environmental filters such as soil composition, topography, or disturbance patterns that occur within grid cells, posing an important question on the point at which global or regional scale processes override fine scale processes on individual sites. To improve our mechanistic understanding and predict the impact of multiple disturbances on microbial communities and their taxonomic, functional, and metabolic attributes, it will be essential to design realistic experimental approaches prioritizing those factors that are most likely to exert a strong influence on microbial communities, using existing knowledge of global change (e.g. factorial experiments considering drought, warming, and heatwaves). Conducting such experiments in a coordinated fashion across different bioregions, where multiple indicators, including taxonomic composition, functional genes and ecosystem processes, are measured using standardized protocols, is also necessary to obtain a better understanding of the impact of multiple disturbances on microbial communities at a global scale and define the role of local conditions in modulating these interactions. This would involve comparing and contrasting the responses of microbial communities to disturbances across different regions, climates, and ecosystems. By doing so, we could assess the responsiveness of each metric at different spatial extents and identify common patterns and mechanisms that operate across different systems and understand how these are influenced by contextual variables such as land use, management practices, and biotic interactions. Given the fundamental role of microorganisms in supporting dryland functioning, it is essential to expand our knowledge on the impact of both global and local drivers of change simultaneously to gain a better understanding of how microbial communities respond to changing environmental conditions and assess how these responses affect dryland functioning, and their vulnerability to degradation.

## Conclusions and future directions

Microorganisms are major players in drylands functioning with a key role in biogeochemical cycling of elements essential for life [57]. Although our understanding of biodiversity and the ecological and physiological attributes of these fascinating organisms is rapidly progressing, several outstanding questions and issues remain. At first glance, a major knowledge gap in drylands microbial ecology is the extent of diversity and adaptation of microbial communities in both soil and lithic substrates. Furthermore, we have very limited understanding of physiological, ecological, and evolutionary mechanisms that underpin adaptations of microbes to their environments. We envisage that linking microbial traits with evolutionary fitness and ecological dynamics through a trait-based approach will make it possible to better understand mechanisms driving microbial adaptation and coexistence across niches [20]. Given the role of microorganisms in mediating feedback mechanisms to climate and anthropogenic change, extending our knowledge on the functional attributes of these communities will be of paramount importance in predicting the impact of future climate change on these fragile and vulnerable communities.

Overall, the emerging body of literature focused on changes in aridity and climatic patterns points to a shift in the distribution of microbial groups linked to soil stability, vegetation, and biogeochemical cycling across the whole aridity spectrum. However, significant knowledge gaps, highlighted above, still exist in our understanding on the potential consequences of these processes and dryland functioning. In particular, empirical data connecting soil microbial diversity and ecosystem functions across large environmental gradients and how regional processes and climate change can affect this relationship are lacking. The interplay between taxonomic composition, functional attributes, and environmental conditions holds the key to unraveling the mechanisms underlying microbial responses to global change drivers. This knowledge will enable us to identify pivotal transition points, assess the vulnerability of ecosystems, and develop effective strategies for conservation and management. Expanding research efforts to bridge knowledge gaps and promote interdisciplinary collaborations will pave the way for a comprehensive understanding of dryland microbiomes and their role in sustaining ecosystem functioning in the face of environmental challenges. Furthermore, the consequences of CO_2_ fertilization and vegetation greening for dryland microorganisms should be also considered.

In-depth studies on dryland microorganisms are needed to assess the effectiveness of biotechnological innovations in reverting climate- and human-driven issues, such as land degradation and desertification. For example, recent research has demonstrated the potential of utilizing biocrusts-associated cyanobacteria as biofertilizers for large-scale dryland restoration efforts [58]. Their ability to fix C and N, improve soil aggregation, and provide a favorable microhabitat for the colonization of heterotrophic microbial communities and later-successional biocrusts species makes them particularly promising [59]. However, the effectiveness of these techniques will likely depend on the type of disturbance, soil type, climate conditions, and the ability to overcome technical limitations related to laboratory cultivation, large-scale production, and field-scale application. Similarly, harnessing the unique genetic resources and stress adaptations provided by dryland microorganisms can also alleviate the negative effects of current agricultural practices. Emerging evidence shows that microbial communities from dry environments might mediate plant adaptation to drought [60]. Current and future assessments of the structure and function of dryland microbiomes, coupled with the development of significant approaches to increase their cultivability, will aid the development of effective and rationally designed microbial-based technologies for sustainable agriculture and forestry in these widespread ecosystems.

**Figure 4:**
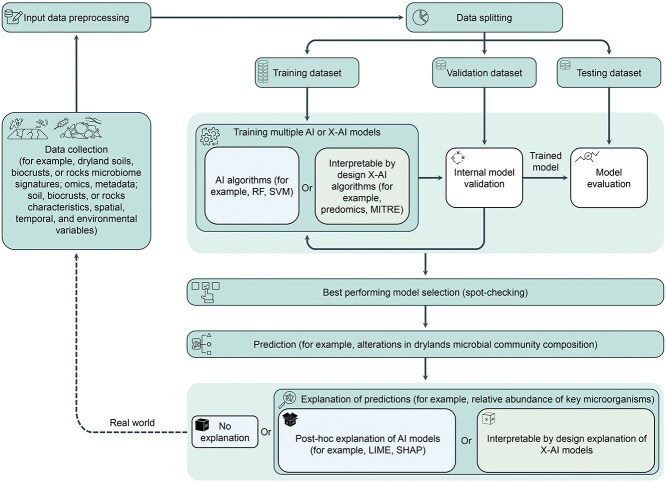
A hypothetical example of applying AI to microbiome research; for instance, for searching for microbial signatures to predict alterations in microbial communities in dryland soils, biocrusts, or rock samples; real-world data are converted to a clean dataset to be used as input for the algorithm through data preprocessing; data are then split into three subsets: training dataset used to fit the model, validation dataset for internal validation of the model where its accuracy is estimated and hyper parameters are tuned, and testing dataset to provide an unbiased evaluation of the final model; multiple AI algorithms such as random forest and support vector machine or interpretable by design X-AI algorithms such as predomics and MITRE can be trained at the same time using the same dataset; a model from the best-performing algorithm is selected through spot-checking to make predictions; producing explanations revolve around two approaches: using post hoc algorithms like Shapley additive explanations (SHAP) or local interpretable model-agnostic explanations to open the black box by identifying important patterns and features underlying their predictions, or using interpretable by design models to explain their own predictions.

We also anticipate that remote sensing and AI will play a central role in advancing dryland microbiomes research (Fig. 1B). Remote sensing could prove to be a valuable tool in studying the composition, structure, and functioning of these ecosystems, using, for instance, high-resolution satellites (e.g. Sentinel-2A/B multispectral imaging twin satellites with signals at 10 m/pixel), miniature satellites (e.g. NASA biological CubeSat, BioSentinel), and drones equipped with sensors that reach a spatial resolution of <1 m/pixel. AI presents a powerful means to derive scientific insights from dryland microbiome data due to its ability to discover intricate patterns in large, highly complex datasets at an accelerated pace (see hypothetical example, Fig. 4). Moreover, with the growing availability of meta-omics data, the integration of these datasets becomes crucial for a comprehensive understanding of microbial community composition and functions. This integration not only holds potential in combining multi-omics data with environmental measures but also demonstrates promise in simultaneously exploring interactions across multiple dimensions of data [61–63].

**Figure Box 1:**
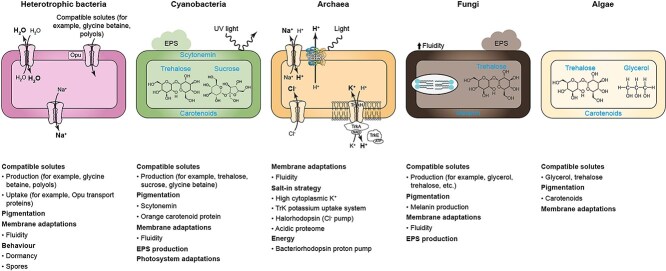
Major molecular and cellular adaptations of dryland microorganisms; the figure depicts examples of the main adaptation strategies (compatible solutes, pigmentation, membrane adaptations, extracellular polymeric substance production, salt-in strategy, photosystem adaptations, energy, and behavior) of dryland microorganisms according to their taxonomic classification (heterotrophic bacteria, cyanobacteria, archaea, fungi, and algae); Cl^−^, chloride anion; H^+^, hydrogen ion; H_2_O, water; Na^+^, sodium ion; NAD, nicotinamide adenine dinucleotide; K^+^, potassium ion; Trk, potassium uptake system, including TrkA, TrkE, and TrkH proteins.

These breakthroughs in the field will change policymakers’ value of science in dryland ecosystems, including hyperarid regions that are often neglected, underestimated, and overlooked. Core to the goal of these scientific explorations is the opportunity to guide future global decision-making policies on drylands biodiversity conservation and monitoring involving key stakeholders such as the UN Convention dealing with Land and Drought, and the Intergovernmental Panel on Climate Change. All these endeavors in drylands microbiome research are expected to lead us to a new age of holistic understanding of microbial life, develop innovative and desirable solutions for stemming biodiversity loss, shine the spotlight on the importance of this field, and ultimately understand and harness the power of the most abundant natural resources on our planet.

## Author contributions

Claudia Coleine, Eleonora Egidi, Manuel Delgado-Baquerizo, Jocelyne DiRuggiero, Brajesh K. Singh, Laura Selbmann, and Antoine L. Harfouche formulated the review and identified the themes to be covered, with contributions from all co-authors. Claudia Coleine, Eleonora Egidi, Jocelyne DiRuggiero, and Antoine L. Harfouche developed and designed the figures. All authors contributed to the first draft of the manuscript and edited the article before submission.

## Conflicts of interest

The authors declare that they have no competing interests.

## Funding

C.C. is supported by the European Commission under the Marie Sklodowska-Curie Grant Agreement No. 702057 (DRYLIFE). A.L.H. is supported by the Italian Ministry of University and Research Brain Gain Professorship and by the European Union Next-Generation EU (Piano Nazionale di Ripresa e Resilienza (PNRR) – Missione 4 Componente 2, Investimento 1.4 – D.D. 1032 17/06/2022, CN00000022) within the Agritech National Research Centre for Agricultural Technologies. M.D-B. acknowledges support from TED2021-130908B-C41/AEI/10.13039/501100011033/ Unión Europea NextGenerationEU/PRTR and from the Spanish Ministry of Science and Innovation for the I + D + i project PID2020-115813RA-I00 funded by MCIN/AEI/10.13039/501100011033. J.D.R and C.P-F. acknowledge funding from the National Aeronautics and Space Administration (NASA) grants 80NSSC19K0470 and NNX15AP18G. E.G. is supported by the European Research Council grant agreement 647038 (BIODESERT) and the Consellería de Educación, Cultura y Deporte de la Generalitat Valenciana, and the European Social Fund (APOSTD/2021/188). B.K.S. acknowledges funding from the Australian Research Council (DP210102081; DP230101448) for microbiome research. E.E. is supported by an Australian Research Council Discovery Early Career Researcher Awards (DECRA) fellowship (DE210101822).

## Data availability

Data sharing is not applicable to this article as no datasets were generated or analyzed during the current study.

## Ethics approval and consent to participate

Not applicable.

## Consent for publication

All authors consent to publication of this article.
