# Correction to: Dryland microbiomes reveal community adaptations to desertification and climate change

**DOI:** 10.1093/ismejo/wraf060

**Published:** 2025-04-15

This is a **correction** to:

Claudia Coleine, Manuel Delgado-Baquerizo, Jocelyne DiRuggiero, Emilio Guirado, Antoine L Harfouche, Cesar Perez-Fernandez, Brajesh K Singh, Laura Selbmann, Eleonora Egidi, Dryland microbiomes reveal community adaptations to desertification and climate change, *The ISME Journal*, Volume 18, Issue 1, January 2024, wrae056, https://doi.org/10.1093/ismejo/wrae056

Funding support to the first author from the European Commission under the Marie Sklodowska-Curie Grant Agreement No. 702057 (DRYLIFE) was omitted from the originally published version.

This error has been corrected.

